# Comparative Evaluation of Ashwagandha (*Withania somnifera*) Root Extract and Melatonin for Improving Sleep Quality in Adults: A Prospective, Randomized, Double-Blind, Placebo-Controlled Study

**DOI:** 10.3390/clockssleep8020015

**Published:** 2026-03-27

**Authors:** Navya Movva, Jaising Salve, Kalpana Wankhede, Vaishali Thakare, Deepak Langade

**Affiliations:** 1 Department of Pharmacology, DY Patil University School of Medicine, Nerul, Navi Mumbai 400706, India; movvanavya444@gmail.com (N.M.); vaishali.thakare@dypatil.edu (V.T.); 2 Department of Internal Medicine, Prakruti Care Hospital, Navi Mumbai 400605, India; 3 Department of General Medicine, Bharati Vidyapeeth Deemed University Dental College & Hospital, Navi Mumbai 400614, India

**Keywords:** Ashwagandha, *Withania somnifera*, melatonin, sleep disturbance, HAM-A, PSQI

## Abstract

Ashwagandha, a revered herb in Ayurvedic medicine for over 3000 years, is recognized for its potential benefits in regulating sleep and supporting overall vitality. This study evaluated the comparative effects of Ashwagandha root extract (ARE) and melatonin (MLT) on sleep quality in adults. In this prospective, randomized, double-blind, placebo-controlled trial, 200 men and women aged 18–50 years were randomized to receive ARE (300 mg twice daily; *n* = 50), MLT (3 mg/day; *n* = 50), a combination of ARE (600 mg/day) and MLT (3 mg/day; *n* = 50), or placebo (*n* = 50) for eight weeks. The primary outcome was the change in sleep onset latency (SOL) from baseline to week eight, measured by actigraphy. Secondary outcomes included actigraphy-based changes in total sleep time (TST), wake after sleep onset (WASO), and sleep efficiency (SE), as well as subjective measures such as the Pittsburgh Sleep Quality Index (PSQI) and the Hamilton Anxiety Scale (HAM-A). At week eight, SOL was significantly reduced across treatment groups, with the ARE–MLT (*p* < 0.0001) combination showing the greatest improvement. The combination group also demonstrated significant improvements in TST (*p* < 0.0001), WASO (*p* < 0.0001), and SE (*p* < 0.0001), whereas ARE and MLT monotherapy produced moderate but comparable benefits. Inferential analyses confirmed statistically significant improvements in objective and subjective sleep measures (*p* < 0.0001). Safety analyses indicated that mild adverse events occurred across all groups, with no clinically significant between-group differences. Overall, both Ashwagandha and melatonin improved sleep disturbances in adults, with combination therapy producing the most consistent and pronounced benefits.

## 1. Introduction

Sleep is an essential part of life that helps restore energy, maintain metabolic balance, and support overall health. Sleep disturbance is common across many countries, affecting roughly 11% to 50% of people in both Western and Asian regions [1]. It includes problems such as trouble falling asleep, waking up often during the night, waking too early, or not feeling refreshed after sleep. These issues can lead to daytime tiredness, irritability, low energy, difficulty concentrating, and a noticeable decline in quality of life [2].

Persistent sleep disturbance that occurs without any medical, emotional, or environmental cause affects about 10–15% of adults [3]. In other cases, sleep problems happen because of underlying physical or mental health conditions [4]. Sleep disturbances also carry broader social and economic consequences, such as more missed workdays, reduced productivity, increased healthcare use, and a higher risk of workplace or traffic accidents [5]. Epidemiological data show that the prevalence of sleep disturbance rises with age and is impacted by characteristics including female gender, reduced socioeconomic status, and concomitant medical conditions [6].

Originally, plant-based remedies have been beneficial for improving sleep quality [7], with Ashwagandha (*Withania somnifera*) emerging up as a versatile adaptogenic herb [8]. Ashwagandha is used to modulate the hypothalamic-pituitary-adrenal (HPA) axis and neuroendocrine functions, which promote physiological homeostasis and stress resilience [9]. Unlike conventional sedatives, which frequently mask symptoms, Ashwagandha reduces the underlying stress and anxiety associated with sleep disturbances. Its rejuvenating and nervine effects promote restorative sleep while also enhance overall vitality. Preclinical research has highlighted its anti-anxiety, anti-inflammatory, and mood-stabilizing effects [10], but clinical evidence on its efficacy in treating sleep disturbance is limited, necessitating rigorous investigations to establish its therapeutic potential.

Melatonin, an endogenous hormone produced in the pineal gland, regulates circadian rhythms and the sleep-wake cycle [11,12]. It is widely used as an over-the-counter treatment for jet lag, delayed sleep onset, and circadian rhythm irregularities. Melatonin-controlled-release formulations have shown potential in enhancing sleep initiation and duration, especially in elderly individuals, where melatonin levels are naturally low [13]. However, systematic reviews and meta-analyses have reported variable efficacy in broader populations, emphasizing the need for tailored dosing and formulation strategies. Aside from its role in sleep regulation, melatonin has antioxidant, anti-inflammatory, and neuroprotective properties, adding to its therapeutic value [14].

Melatonin was selected as a comparator in this study because it is a well-established, evidence-supported, and commonly used natural agent for sleep regulation [15]. Its chrono-biotic action on circadian alignment and sleep initiation [16] provides a clear mechanistic contrast to Ashwagandha, which primarily acts through stress reduction and HPA-axis modulation [9]. Using melatonin as a benchmark enables direct comparison between two widely used but mechanistically distinct natural sleep aids, allowing assessment of their relative efficacy, potential complementary benefits. Thus, the present study investigated the synergistic effects of standardized Ashwagandha root extract (ARE) and melatonin (MLT) in improving the sleep quality in adults. This randomized, double-blind, placebo-controlled trial assessed the combined efficacy of Ashwagandha’s adaptogenic and restorative properties and melatonin’s chrono-biotic effects in improving sleep parameters and overall quality of life in adults.

This study evaluated the effects of standardized Ashwagandha root extract, melatonin, and their combination on objective and subjective sleep outcomes in adults with sleep disturbances. The primary objective was to determine whether combined administration produced greater improvements in actigraphy-derived sleep onset latency, total sleep time, wake after sleep onset, and sleep efficiency compared with monotherapy or placebo. Secondary objectives included assessment of subjective sleep quality, mental alertness on rising, anxiety-related outcomes, and safety. It was hypothesized that the combined intervention would result in superior improvements in sleep parameters due to complementary modulation of circadian and stress-related mechanisms, while Ashwagandha and melatonin administered individually would demonstrate meaningful benefits relative to placebo without compromising tolerability.

## 2. Results

### 2.1. Participant Disposition

A total of 242 participants were assessed for eligibility. Of these, 23 did not meet the eligibility criteria and 19 declined participations. The remaining 200 participants were enrolled and randomized equally into four groups: ARE (*n* = 50), MLT (*n* = 50), ARE–MLT (*n* = 50), and PL (*n* = 50).

**Figure 1:**
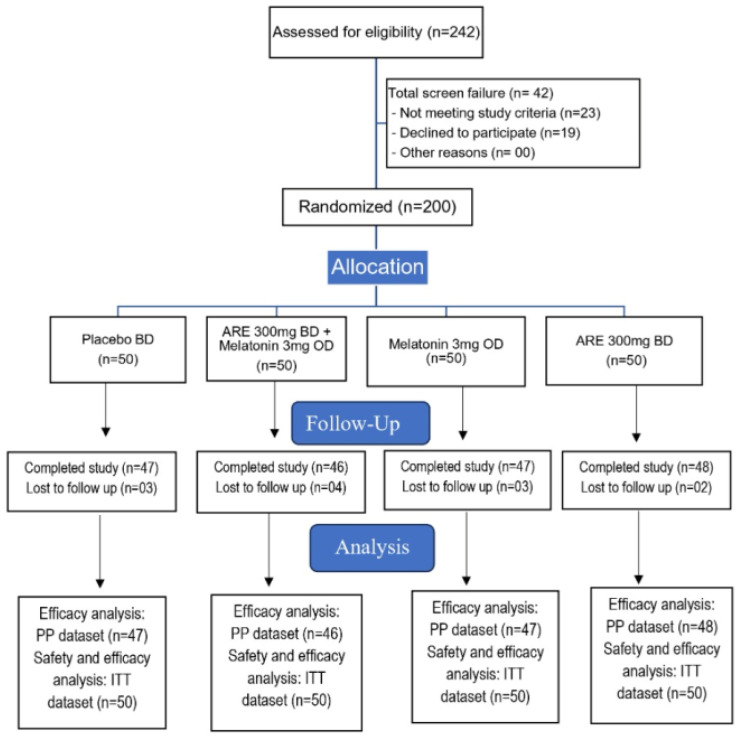
CONSORT patient flow diagram.

During the study, 12 participants were lost to follow-up (ARE: 02; MLT: 03; ARE–MLT: 04; PL: 03). Thus, 188 participants were included in the per protocol (PP) analysis, while all 200 participants were included in the intention to treat (ITT) analysis for safety and efficacy outcomes. Participant disposition is illustrated in Figure 1 (CONSORT flow diagram).

### 2.2. Baseline Characteristics

Baseline demographic and clinical characteristics were comparable across the four groups (*p* > 0.05). There were no significant differences in age, body mass index, or occupation. Similarly, baseline sleep parameters, including SOL, TST, WASO, TIB, SE, and subjective scores (HAM-A, PSQI, MARS, sleep quality) were comparable across groups (*p* > 0.05, Table 1).

### 2.3. Effect of Interventions on Sleep Onset Latency, Total Sleep Time, WASO, and Time in Bed

**Figure 2:**
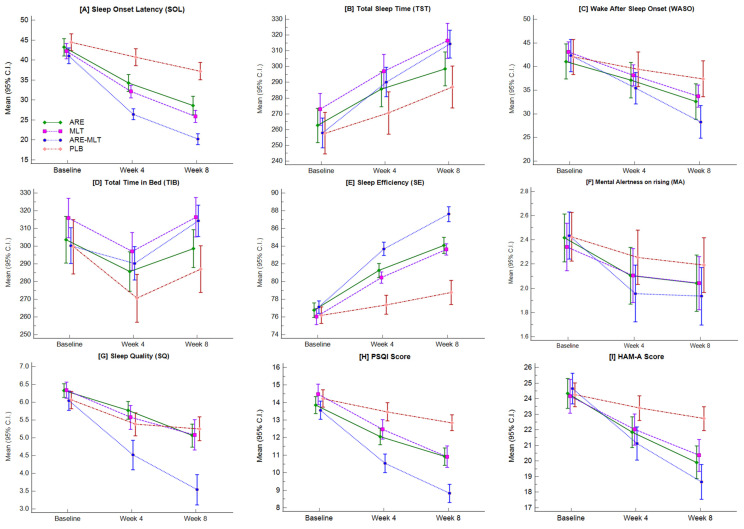
Mean actigraphy-based sleep outcomes measured from baseline to week 4 and week 8 in the per-protocol dataset. The plots illustrate: (**A**) Sleep onset latency (SOL), (**B**) Total sleep time (TST), (**C**) Wake after sleep onset (WASO), (**D**) Total Time in Bed (TIB), (**E**) Sleep efficiency (SE), (**F**) Mental alertness on rising (MA), (**G**) Sleep quality (SQ), (**H**) PSQI score, and (**I**) HAM-A score.

Actigraphy assessments revealed significant improvements from baseline for all sleep parameters in the active treatment groups compared with placebo, as shown in Table 2 and Figure 2.

Sleep Onset Latency (SOL): At week four, SOL decreased significantly in all active groups: ARE −8.9 ± 2.8 min, MLT −10.1 ± 5.2 min, ARE–MLT −14.6 ± 4.3 min, vs. PLB −3.7 ± 1.0 min; ANOVA F (3, 184) = 68.66, *p* < 0.0001. At week eight, reductions were ARE −14.6 ± 3.4 min, MLT −16.4 ± 5.3 min, ARE–MLT −20.9 ± 4.2 min, vs. PLB −7.2 ± 1.8 min; ANOVA F (3, 184) = 97.75, *p* < 0.0001. Post hoc Bonferroni tests indicated that ARE–MLT produced significantly greater reductions in SOL compared with ARE (*p* < 0.01), MLT (*p* < 0.01), and PLB (*p* < 0.001).

Total Sleep Time (TST): Week 4 increases were ARE +23.0 ± 13.2 min, MLT +24.1 ± 8.7 min, ARE–MLT +32.3 ± 0.9 min, PLB +12.9 ± 2.8 min; ANOVA F (3, 184) = 44.82, *p* < 0.0001. At week eight, TST improvements were ARE +35.9 ± 8.7 min, MLT +43.5 ± 12.5 min, ARE–MLT +56.3 ± 3.7 min, PLB +29.3 ± 3.4 min; ANOVA F (3, 184) = 96.34, *p* < 0.0001. Post hoc comparisons confirmed that ARE–MLT yielded the largest increase compared with all other groups (*p* < 0.01).

Wake After Sleep Onset (WASO): Week 4 reductions were ARE −4.0 ± 1.1 min, MLT −4.9 ± 4.1 min, ARE–MLT −6.9 ± 5.0 min, PLB −2.6 ± 1.4 min; ANOVA F (3, 184) = 13.74, *p* < 0.0001. Week 8 reductions were ARE −8.5 ± 1.5 min, MLT −9.3 ± 6.0 min, ARE–MLT −14.0 ± 8.4 min, PLB −4.6 ± 1.9 min; ANOVA F (3, 184) = 25.03, *p* < 0.0001. Post hoc analysis indicated ARE–MLT significantly outperformed ARE, MLT, and PLB (*p* < 0.05).

Time in Bed (TIB): At week four, increases were ARE +19.0 ± 13.1 min, MLT +19.2 ± 7.2 min, ARE–MLT +25.4 ± 5.1 min, PLB +10.3 ± 2.6 min; ANOVA F (3, 184) = 27.75, *p* < 0.0001. At week eight, TIB increased by ARE +27.4 ± 8.7 min, MLT +34.1 ± 9.9 min, ARE–MLT +42.3 ± 7.5 min, PLB +24.7 ± 3.3 min; ANOVA F (3, 184) = 47.69, *p* < 0.0001. ARE–MLT demonstrated the largest gain compared with other groups (*p* < 0.01).

Effect sizes (partial η^2^) for week 8 improvements were large across all sleep log parameters: SOL = 0.61, TST = 0.61, WASO = 0.29, TIB = 0.44, respectively.

### 2.4. Effect on Sleep Log Parameters

**Figure 3:**
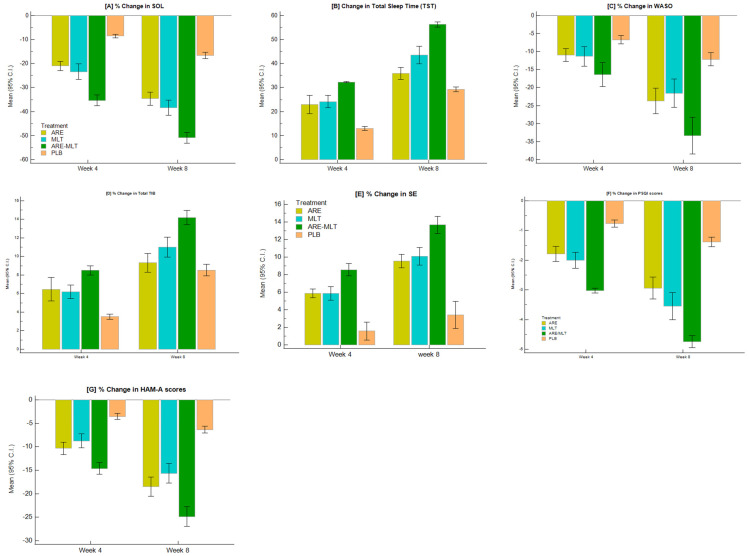
Percentage change in mean sleep log actigraphy outcomes, subjective sleep measures, and psychological assessments from baseline in the PP dataset. Shown are % changes for: (**A**) Sleep onset latency (SOL), (**B**) Total sleep time (TST), (**C**) Wake after sleep onset (WASO), (**D**) Total time in bed (TIB), (**E**) Sleep efficiency (SE), (**F**) PSQI scores, and (**G**) HAM-A scores.

The effects of all the Sleep Log Parameters are depicted in Table 3 and Figure 3.

Sleep Efficiency (SE): Significant week 4 improvements were ARE +4.5 ± 1.2%, MLT +4.4 ± 1.9%, ARE–MLT +6.6 ± 1.7%, PLB +1.2 ± 2.7%; ANOVA F (3, 184) = 59.68, *p* < 0.0001. Week 8 improvements were ARE +7.3 ± 2.0%, MLT +7.6 ± 2.3%, ARE–MLT +10.5 ± 2.4%, PLB +2.6 ± 4.1%; ANOVA F (3, 184) = 64.63, *p* < 0.0001.

MARS: Week 4 changes were not significant (*p* = 0.051), but week 8 showed a significant decrease in ARE–MLT vs. PLB (*p* = 0.006).

Sleep Quality: Week 4 changes were ARE −0.6 ± 0.5, MLT −0.8 ± 0.7, ARE–MLT −1.5 ± 0.8, PLB −0.7 ± 0.5; ANOVA F (3, 184) = 23.03, *p* < 0.0001. Week 8 changes were ARE −1.3 ± 0.7, MLT −1.3 ± 1.0, ARE–MLT −2.5 ± 0.8, PLB −0.8 ± 0.6; ANOVA F (3, 184) = 39.07, *p* < 0.0001.

At week eight, partial eta squared values indicated large treatment effects across sleep efficiency (η^2^ = 0.50), sleep quality (η^2^_p_ = 0.39–0.61), with a moderate effect observed for morning alertness (η^2^_p_ = 0.07).

### 2.5. Effect on Subjective Sleep Parameters (PSQI, HAM-A, SE, MARS, Sleep Quality)

**Figure 4:**
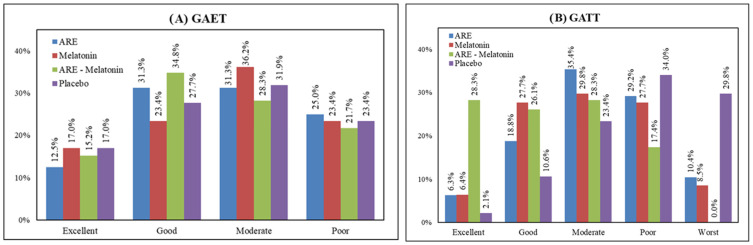
Global Assessment of Efficacy and Tolerability to Therapy (GAET & GATT) in PP dataset (*n* = 188) (**A**) GAET (**B**) GATT.

The effect on subjective sleep parameters such as PSQI, HAM-A, SE, MARS, and Sleep quality is presented in Table 4 and Figure 4

**PSQI**: Baseline scores were comparable (*p* = 0.076). Week 4 reductions were ARE −1.8 ± 0.9, MLT −2.0 ± 0.9, ARE–MLT −3.0 ± 0.3, PLB −0.8 ± 0.4; ANOVA F (3, 184) = 85.78, *p* < 0.0001. Week 8 reductions were ARE −2.9 ± 1.3, MLT −3.6 ± 1.6, ARE–MLT −4.7 ± 0.7, PLB −1.4 ± 0.5; ANOVA F (3, 184) = 75.21, *p* < 0.0001. Post hoc Bonferroni analysis showed ARE–MLT was superior to ARE, MLT, and PLB (*p* < 0.01).

**HAM-A**: Week 4 changes were ARE −2.5 ± 1.0, MLT −2.1 ± 1.3, ARE–MLT −3.5 ± 0.8, PLB −0.9 ± 0.5; ANOVA F (3, 184) = 63.59, *p* < 0.0001. Week 8 changes were ARE −4.4 ± 1.5, MLT −3.8 ± 1.9, ARE–MLT −6.0 ± 1.4, PLB −1.5 ± 0.6; ANOVA F (3, 184) = 78.49, *p* < 0.0001. Post hoc comparisons confirmed ARE–MLT superiority (*p* < 0.01).

At week 8, both PSQI and HAM-A demonstrated large effect sizes (η^2^ = 0.58 and 0.56, respectively).

### 2.6. Global Assessments of Efficacy and Tolerability

Efficacy: The “Excellent” rating was highest in the MLT group (17.8%), whereas “Good” efficacy was most frequent in the ARE–MLT group (34.8%). “Poor” efficacy was highest in the placebo group (34.0%).

Tolerability (GATT): “Excellent” tolerability was highest in the MLT group (17.0%), while “Good” tolerability was most frequent in the ARE–MLT group (34.8%). No participant reported “Worst” tolerability in any group were depicted in Figure 4.

### 2.7. Adverse Events

Overall incidence of adverse events was low and comparable across groups: ARE 6%, MLT 10%, ARE–MLT 12%, PLB 6%. The most common events were nausea (4.5%), mild headache, and abdominal pain (1.5% each). Drowsiness was rare (1%). No serious adverse events were reported.

## 3. Discussion

Adequate sleep is essential for optimal neurocognitive function, learning, memory consolidation, cardiovascular health, metabolic regulation, and overall well-being. Disturbed sleep can lead to physical and mental health issues and is frequently associated with impaired quality of life. Given the limitations and side effects associated with conventional pharmacological treatments [17,18], there is increasing interest in herbal remedies such as Ashwagandha root extract (ARE) for sleep improvement, particularly in adults experiencing stress-related or disturbed sleep [19,20,21]. The primary objective of this study was to evaluate the efficacy and safety of ARE alone, melatonin (MLT) alone, and their combination (ARE–MLT) in improving objective and subjective sleep outcomes over eight weeks compared with placebo.

### 3.1. Sleep Onset and Sleep Duration

The present study demonstrated significant improvements in sleep onset latency (SOL) and total sleep time (TST) in all active treatment groups relative to placebo. Notably, the combination therapy (ARE–MLT) showed the largest reduction in SOL (−20.9 ± 4.2 min) and the greatest increase in TST (Week 8: +56.3 ± 3.7 min), followed by MLT and ARE alone. These findings are consistent with previously reported studies by Langade et al., (2019) and Salve et al. (2019), indicating that Ashwagandha modulates the hypothalamic-pituitary-adrenal axis and reduces cortisol levels, mitigating stress-induced hyperarousal that interferes with sleep initiation [8,20]. Melatonin, by contrast, primarily regulates circadian rhythms, facilitating sleep onset. The synergistic effect observed in the ARE–MLT group may be explained by complementary mechanisms: ARE reduces physiological arousal and stress, while melatonin promotes circadian alignment.

### 3.2. Sleep Maintenance and Efficiency

Wake after sleep onset (WASO) and sleep efficiency (SE) also improved significantly in all treatment groups. The ARE–MLT combination reduced WASO by 14 min, compared with 9.3 min in the MLT group and 8.5 min in the ARE group. Sleep efficiency improvements mirrored these trends. These outcomes align with recent studies reported by Langade et al. (2021) and Atul et al. (2020), indicating that Ashwagandha enhances GABAergic and serotonergic signaling, contributing to sustained sleep duration and quality [21,22]. Actigraphy-based measures provided objective evidence supporting these improvements, strengthening the validity of our findings.

### 3.3. Subjective Sleep Quality and Anxiety

Subjective assessments using the Pittsburgh Sleep Quality Index (PSQI), sleep logs, and HAM-A scores showed significant reductions in sleep disturbances and anxiety across all active groups. The combination therapy produced the largest decreases in PSQI (−4.7 ± 0.7) and HAM-A scores, whereas ARE and MLT alone achieved moderate but significant improvements. These results are in line with earlier studies reported by Langade et al. (2019), Xu et al. (2020), and Pachikian et al. (2021), highlighting the anxiolytic properties of Ashwagandha and its role in enhancing sleep quality [20,23,24]. The combination therapy demonstrated additive benefits, supporting the rationale for dual-target interventions addressing both sleep physiology and stress modulation.

### 3.4. Safety and Tolerability

All interventions were well-tolerated. The most common adverse events were mild nausea and drowsiness, primarily in the ARE–MLT and MLT groups. No serious adverse events were reported, and the majority of participants rated efficacy and tolerability as “good” or “excellent.” These findings are consistent with prior clinical trials of Ashwagandha and melatonin, suggesting that both interventions are safe and suitable for adult populations with sleep disturbances.

### 3.5. Comparison with Recent Literature

Our findings are largely consistent with updated literature over the past five years. Recent randomized controlled trials have demonstrated the efficacy of ARE in improving both objective and subjective sleep parameters in adults with insomnia or stress-related sleep disturbance [20,22]. Similarly, combination strategies targeting multiple pathways, including circadian alignment and stress reduction, have been recommended to optimize treatment outcomes. While melatonin alone is effective in sleep onset, its combination with ARE provides a broader spectrum of benefits, including enhanced sleep maintenance, efficiency, and reduced anxiety, supporting clinical use of this dual approach [25,26,27].

### 3.6. Strengths

This study has several strengths. The prospective, randomized, double-blind, placebo-controlled design ensures robust control over treatment conditions and minimizes bias. The use of actigraphy provides objective, high-resolution sleep measures, complementing subjective assessments. In addition to this, the study isolates both individual and combined effects of ARE and MLT, offering insight into the relative and synergistic benefits of these interventions.

### 3.7. Limitations

Several limitations should be acknowledged. Sleep outcomes may be influenced by environmental factors, including noise, light exposure, and daily activity levels, which were not fully controlled. Self-reported measures, including PSQI, sleep logs, and HAM-A, are subject to recall bias and subjective interpretation. The sample size, while adequate for primary outcomes, may limit generalizability to broader populations. Future studies should consider longer follow-up, objective biomarkers of sleep and stress, and diverse demographic cohorts to validate and extend these findings.

### 3.8. Clinical Implications and Future Directions

The present study provides evidence that ARE, MLT, and especially their combination, are effective, safe, and well-tolerated interventions for improving sleep quality and reducing anxiety in adults. The synergistic benefits of ARE–MLT highlight the potential for combination therapy targeting both physiological arousal and circadian regulation. Future research should explore long-term outcomes, dose optimization, and applicability to populations with chronic insomnia or comorbid conditions. Clinically, these findings support the integration of herbal and circadian-based interventions as complementary strategies for sleep management

## 4. Materials and Methods

### 4.1. Study Design and Setting

This study evaluated the efficacy of Ashwagandha root extract combined with melatonin for improving sleep disturbance in adults using a randomized, double-blind, placebo-controlled trial. The independent variable was the treatment assignment, with participants randomized to receive ARE (300 mg twice daily), MLT (3 mg/day), a combination of ARE + MLT, or placebo. The dependent variables included objective sleep measures: Sleep onset latency (SOL), total sleep time (TST), wake after sleep onset (WASO), and sleep efficiency (SE), assessed via actigraphy, as well as subjective outcomes including Pittsburgh Sleep Quality Index (PSQI) and Hamilton Anxiety Scale (HAM-A) scores. Safety outcomes, such as adverse events, were also monitored.

The study protocols and associated documents were approved by the Institutional Ethics Committee of D. Y. Patil Medical College & Hospital, Navi Mumbai, India (IEC Reference No.: DYP/IEC/23/2022). The study followed ICH GCP guidelines, India’s New Drugs and Clinical Trials Rules 2019, the Declaration of Helsinki, and other relevant regulations. Written informed consent was obtained from all participants before conducting study-related activities. The study is registered with the Clinical Trials Registry of India (CTRI/2022/10/046179). The reporting of the study’s findings adheres to the CONSORT (Consolidated Standards of Reporting Trials) guidelines. The treatment period for the study was initially four weeks but was extended to eight weeks based on expert and ethics committee recommendations. This extension improved safety monitoring and accurately reflected therapy duration.

### 4.2. Participants

#### 4.2.1. Sample Size Calculation

As epidemiological data from the region were not available, this study was conducted as a pilot study. The sample size was based on changes in sleep onset latency (SOL) reported in a 5-week randomized, placebo-controlled study of melatonin (5 mg/day) by Smits M.G. et al. (2016) [26], which reported mean (SD) SOL changes of 28.4 (30.15) for melatonin and 12.1 (32.8) for placebo. Assuming a 10% greater improvement with combination therapy of melatonin and Ashwagandha, 42 participants per group (total n = 168) would provide 90% power to detect differences among group means using one-way ANOVA at a 0.05 significance level. To account for potential data loss, an additional 32 participants were included, resulting in a total sample of 200 participants, with 50 in each group.

#### 4.2.2. Randomization and Blinding

Enrolled participants (*n* = 200) were assigned unique serial numbers and randomized in blocks of 40, ensuring equal distribution across the four treatments: ARE (*n* = 50), MLT (*n* = 50), combination therapy of ARE–MLT (*n* = 50), or PL (*n* = 50) (Figure 1). Pre-determined computer-generated randomization was used to allocate participants to treatment groups. Study dose packs were pre-sealed and distributed based on the participants’ serial numbers, ensuring an identical appearance for all investigational products, including placebo capsules. Blinding was maintained using sequentially numbered opaque sealed envelopes (SNOSE) containing the randomization codes, which were securely held by the principal investigator. Neither the participants nor the research team, including investigators and site staff, were aware of the treatment allocations. The randomization codes could only be accessed by the investigator in case of an emergency. Study assessments at each visit were conducted by a non-affiliated researcher who was also blinded to the treatment groups.

#### 4.2.3. Eligibility Criteria

Inclusion criteria:

1. The study enrolled 200 male and female adults aged 18 to 50 years who met the DSM-5 [27] diagnostic criteria for sleep disturbance.
2. Eligible participants reported difficulty falling asleep (sleep latency > 30 min), a total sleep time of ≤6.5 h per night (at least three nights per week) and associated daytime complaints.
3. Participants were required to have a habitual bedtime between 8.30 PM and midnight and a body mass index (BMI) between 16.5 and 30 kg/m^2^.
4. Participants needed to keep a sleep diary, follow study procedures, and provide written informed consent.

Exclusion criteria:

1. Participants were excluded if they were pregnant or breastfeeding women. Individuals with significant endocrine, metabolic, hepatic, renal, cardiovascular, gastrointestinal, respiratory, hematological, or neurological conditions, as well as those with psychiatric disorders contributing to insomnia, were not eligible.
2. Other exclusions included the presence of sleep disorders, such as restless leg syndrome or sleep apnea, substance abuse within the past year, or tobacco use during night awakenings.
3. Additional exclusions applied to individuals with a history of seizures or significant head trauma, recent travel across four or more time zones, or shift work within seven days prior to the study.
4. Regular use of medications (excluding antihypertensives, antidiabetics, lipid-lowering agents, and primary cardiovascular prophylactics), recent participation in investigational drug trials, or an inability to comply with study procedures or unwilling to provide written informed consent were excluded.

### 4.3. Interventions

Participants in the combination group (ARE–MLT) received a capsule containing 300 mg of KSM-66 Ashwagandha standardized root extract (Ixoreal Biomed Inc., Los Angeles, CA, USA) administered twice daily after breakfast and dinner, along with a 3 mg melatonin tablet once daily after dinner, for a duration of eight weeks. Participants in the ARE group were given a 300 mg capsule of KSM-66 Ashwagandha standardized root extract with a placebo tablet identical to the melatonin tablet. The MLT group received a 3 mg melatonin tablet once daily after dinner together with a placebo capsule identical to the Ashwagandha capsule. The placebo group (PL) received both a placebo capsules identical to the Ashwagandha capsule and a placebo tablet identical to the melatonin tablet.

### 4.4. Investigational Product Details

The investigational product KSM-66 Ashwagandha was obtained from the manufacturer Ixoreal Biomed Inc., Los Angeles, CA, USA. KSM-66 is the commercially available highest concentration root-only extract of Ashwagandha, which is produced through a green chemistry method that is devoid of alcohol or any chemical solvents. The product contains a root-only extract from Ashwagandha, with an optimum amount of withanolides (>5%) precisely estimated by the High-Performance Liquid Chromatography (HPLC) method.

Melatonin was provided as 3 mg oral tablets. Placebo capsules (starch) and placebo tablets were manufactured to be identical in color, shape, and size to the active products to maintain blinding. Thus, the participants in each group received capsules and tablets that were visually indistinguishable across treatment arms.

### 4.5. Study Outcomes

The primary outcome was the change in sleep onset latency (SOL) from baseline to week 8, measured by actigraphy. Secondary outcomes included actigraphy-based changes in total sleep time (TST), wake after sleep onset (WASO), and sleep efficiency (total time in bed, TIB). Other secondary outcomes were changes in Pittsburgh Sleep Quality Index (PSQI) score (number and duration of sleep interruptions, quality of sleep, daytime naps, and nature of waking activities), and mental alertness on rising (MARS), all assessed using a sleep log. Changes in the Hamilton Anxiety Scale (HAM-A) total score were also evaluated. Safety assessments included monitoring adverse events and laboratory parameters throughout the study period. These outcomes provided a comprehensive assessment of the efficacy and safety of the interventions in improving sleep quality and associated parameters.

### 4.6. Study Assessments

#### 4.6.1. Objective Sleep Assessment (Actigraphy)

Sleep parameters were assessed by using actigraphy to objectively monitor sleep-wake patterns. Participants wore an Actiwatch 2 device (Respironics, 1001, Murry Ridge Lane, Murrysville, PA, USA) on the non-dominant wrist, which continuously recorded movement for a minimum of three consecutive days. Data was processed using Actiware software (version 6.0). Key sleep parameters extracted included sleep onset latency (SOL), total sleep time (TST), wake after sleep onset (WASO), and sleep efficiency (SE), allowing for an objective and comprehensive evaluation of sleep architecture and overall sleep quality.

#### 4.6.2. Pittsburgh Sleep Quality Index (PSQI)

Sleep quality was assessed using the Pittsburgh Sleep Quality Index (PSQI), a widely validated questionnaire that evaluates subjective sleep quality over the preceding month. It consists of seven components: subjective sleep quality, sleep latency, sleep duration, habitual sleep efficiency, sleep disturbances, use of sleep medication, and daytime dysfunction. A global score ranging from 0 to 21 was generated, with higher scores reflecting poorer overall sleep quality [28].

#### 4.6.3. Hamilton Anxiety Rating Scale (HAM-A)

Anxiety severity was evaluated using the Hamilton Anxiety Rating Scale (HAM-A). This scale includes 14 items that assess both psychological symptoms (such as tension and fears) and somatic symptoms (such as restlessness and fatigue). Each item is rated on a 5-point scale from 0 (not present) to 4 (severe). Total scores were interpreted as follows: mild anxiety (<17), mild to moderate anxiety (18–24), and moderate to severe anxiety (≥25) [29].

#### 4.6.4. Mental Alertness on Rising (MARS)

Mental alertness was assessed using the Mental Alertness Rating Scale (MARS), which participants completed each morning. They rated their alertness on a 3-point scale: alert, slightly drowsy, or extremely drowsy, providing a direct measure of next-morning cognitive readiness and the immediate after-effects of sleep quality [22].

#### 4.6.5. Sleep Quality

Sleep quality was evaluated using a seven-point subjective rating scale, completed by participants each morning upon waking. Subjects rated their overall sleep quality using one of the following categories: Excellent, Very Good, Good, Fair, Poor, Very Poor, or Extremely Poor. Assessments were recorded at Screening (Day −3), Baseline (Day 0), Week 1 (±1 day), Week 2 (±2 days), and Week 4 (±2 days) [8].

#### 4.6.6. Safety Assessment

Safety was monitored by recording adverse events (AEs) reported by participants or observed by clinicians during follow-ups, at the time of informed consent, and continuing through study completion. Abnormal clinical or laboratory findings were further evaluated if they required medical intervention.

### 4.7. Statistical Methods and Data Analysis

Data of all participants who received at least one dose of study medication was used for analysis. All relevant statistical calculations were completed using Statistical Package (StataCorp. 2013. Stata: Release 13. Statistical Software. StataCorp LP: College Station, TX, USA), and figures were prepared using the MedCalc Statistical Software, version 23.4.9. 2025 (MedCalc Software bvba, Ostend, Belgium). Measurement data for laboratory and total scores for PSQI, sleep log assessments, sleep actigraphy assessments and HAM-A scales will be presented as means with SD. Categorical data would be analyzed as numbers and percentages (proportions). Ranking data and scores would be calculated and presented as means with SD. 95% confidence intervals (C.I.) would be presented wherever applicable. Change in the scores from baseline would be calculated and expressed as means with SD. Categorical and nominal data will be presented as number with percentages. Baseline values and scores will be compared to post-treatment values and score for the different scales using the repeat measures ANOVA for normally distributed data. Post hoc individual comparisons will be done using *t*-test for normal data and Bonferroni’s multiple comparisons (<0.05). Between-group comparisons will be done using the One-Way ANOVA for normal data. Nominal data will be compared between the groups using the chi-square test. All testing would be done using two-sided tests at alpha 0.05.

## 5. Conclusions

This study evaluated the efficacy and safety of Ashwagandha root extract, melatonin, and their combination for improving sleep in adults. The results show that the combination therapy produced the greatest improvements in sleep onset latency, total sleep time, sleep efficiency, and sleep quality, while also reducing anxiety, compared with ARE or MLT alone. Both ARE and MLT were safe and well-tolerated, with only mild adverse events reported. These findings indicate that ARE, especially when combined with melatonin, is a promising, non-pharmacological intervention for adults with stress-related or primary sleep disturbances, addressing both objective and subjective aspects of sleep. Further studies with larger samples and longer follow-up are needed to confirm these benefits and their clinical practice.

## Figures and Tables

**Table 1:** Baseline profile of participants in four groups (ITT dataset).

Parameters	ARE (*n* = 50)	MLT (*n* = 50)	ARE–MLT (*n* = 50)	PLB (*n* = 50)	ANOVA
	Mean (SD)	Mean (SD)	Mean (SD)	Mean (SD)	*p* *
**Anthropometry**					
Age (yrs.)	39.36 (5.54)	39.96 (6.51)	38.14 (3.13)	38.28 (5.13)	0.244
BMI (kg/m^2^)	26.24 (4.05)	25.06 (3.87)	26.57 (3.39)	26.31 (6.05)	0.339
**Sleep actigraphy assessments**					
SOL (min)	43.22 (7.34)	42.34 (6.42)	41.36 (6.30)	44.24 (7.01)	0.181
TST (min)	263.22 (36.49)	272.90 (33.81)	256.86 (33.25)	256.40 (43.77)	0.095
WASO (min)	41.72 (13.03)	42.62 (7.42)	41.84 (11.24)	41.50 (12.42)	0.963
TIB (min)	304.94 (44.87)	315.52 (37.39)	298.70 (35.16)	297.90 (51.25)	0.145
**Sleep log assessments**					
Sleep Efficiency (%)	76.70 (2.83)	76.21 (3.06)	76.92 (2.45)	76.28 (3.07)	0.547
MARS score	2.42 (0.67)	2.32 (0.65)	2.40 (0.67)	2.46 (0.68)	0.762
Sleep quality	6.36 (0.66)	6.28 (0.78)	6.00 (0.93)	6.06 (0.84)	0.082
**PSQI score**	13.86 (1.70)	14.40 (1.97)	13.58 (1.74)	14.16 (1.68)	0.113
**HAM-A score**	24.28 (3.25)	24.32 (3.71)	24.66 (3.40)	24.06 (2.65)	0.835
	No. (%)	No. (%)	No. (%)	No. (%)	*p* **
**Gender**					
Male	36 (72.0%)	35 (70.0%)	38 (76.0%)	36 (72.0%)	0.927
Female	14 (28.0%)	15 (30.0%)	12 (24.0%)	14 (28.0%)	
**Occupation**					
Housewife	5 (10.0%)	7 (14.0%)	5 (10.0%)	4 (8.0%)	0.342
Teacher	4 (8.0%)	4 (8.0%)	6 (12.0%)	2 (4.0%)	
Self employed	14 (28.0%)	9 (18.0%)	10 (20.0%)	19 (38.0%)	
Service	27 (54.0%)	27 (54.0%)	24 (48.0%)	23 (46.0%)	
Unemployed	0 (0.0%)	3 (6.0%)	5 (10.0%)	2 (4.0%)	

SD: Standard deviation; BMI: Body mass index; MLT: melatonin; PLB: Placebo; SOL: Sleep onset latency; TST: Total sleep time; WASO: Wake after sleep onset; TIB: Total time in bed; ARE: Ashwagandha root extract; PSQI: Pittsburgh Sleep Quality Index; HAM-A: Hamilton Anxiety Rating Scale; MARS: Mental alertness on rising. *p* *: One-way analysis of variance (ANOVA); *p* **: Chi-square test.

**Table 2:** Sleep actigraphy assessments in PP dataset.

	ARE (*n* = 48)	MLT (*n* = 47)	ARE–MLT (*n* = 46)	PLB (*n* = 47)	ANOVA	
	Mean (SD)	Mean (SD)	Mean (SD)	Mean (SD)	F	*p* *
**SOL (min)**						
Baseline	43.2 (7.5)	42.3 (6.6)	41.1 (6.5)	44.4 (7.2)	1.98	0.118
Change from baseline						
Week 4	−8.9 (2.8)	−10.1 (5.2)	−14.6 (4.3)	−3.7 (1.0)	68.66	<0.0001
Week 8	−14.6 (3.4)	−16.4 (5.3)	−20.9 (4.2)	−7.2 (1.8)	97.75	<0.0001
% change from baseline						
Week 4	−20.6%	−23.9%	−35.5%	−8.3%		
Week 8	−33.8%	−38.8%	−50.9%	−16.2%		
**TST (min)**						
Baseline	262.6 (37.0)	272.9 (34.4)	258.0 (31.8)	257.7 (44.9)	1.69	0.172
Change from baseline						
Week 4	23.0 (13.2)	24.1 (8.7)	32.3 (0.9)	12.9 (2.8)	44.82	<0.0001
Week 8	35.9 (8.7)	43.5 (12.5)	56.3 (3.7)	29.3 (3.4)	96.34	<0.0001
% change from baseline						
Week 4	8.8%	8.8%	12.5%	5.0%		
Week 8	13.7%	15.9%	21.8%	11.4%		
**WASO (min)**						
Baseline	41.1 (12.9)	43.1 (7.2)	42.3 (11.4)	42.0 (12.6)	0.26	0.856
Change from baseline						
Week 4	−4.0 (1.1)	−4.9 (4.1)	−6.9 (5.0)	−2.6 (1.4)	13.74	<0.0001
Week 8	−8.5 (1.5)	−9.3 (6.0)	−14.0 (8.4)	−4.6 (1.9)	25.03	<0.0001
% change from baseline						
Week 4	−9.7%	−11.4%	−16.3%	−6.2%		
Week 8	−20.7%	−21.6%	−33.1%	−11.0%		
**TIB (min)**						
Baseline	303.7 (45.4)	316.0 (37.8)	300.3 (34.2)	299.7 (52.3)	1.44	0.232
Change from baseline						
Week 4	19.0 (13.1)	19.2 (7.2)	25.4 (5.1)	10.3 (2.6)	27.75	<0.0001
Week 8	27.4 (8.7)	34.1 (9.9)	42.3 (7.5)	24.7 (3.3)	47.69	<0.0001
% change from baseline						
Week 4	6.3%	6.1%	8.5%	3.4%		
Week 8	9.0%	10.8%	14.1%	8.2%		

* One-way analysis of variance (ANOVA). ARE: Ashwagandha root extract; MLT: melatonin; PLB: Placebo; SD: Standard deviation; SOL: Sleep onset latency; TST: Total sleep time; WASO: Wake after sleep onset; TIB: Total time in bed. Post hoc Bonferroni’s multiple comparisons (*p* < 0.05): **ARE vs. Placebo** (SOL: week 4, week 8, change at week 4 and change at week 8; TST: change in week 4 and change at week 8; WASO: change at week 8; TIB: change at week 4).

**Table 3:** Sleep log assessments in PP dataset.

	ARE (*n* = 48)	MLT (*n* = 47)	ARE–MLT (*n* = 46)	PLB (*n* = 47)	ANOVA	
	Mean (SD)	Mean (SD)	Mean (SD)	Mean (SD)	F	*p* *
**SE (%)**						
Baseline	76.8 (2.8)	76.1 (3.1)	77.1 (2.4)	76.2 (3.1)	1.39	0.246
Change from baseline						
Week 4	4.5 (1.2)	4.4 (1.9)	6.6 (1.7)	1.2 (2.7)	59.68	<0.0001
Week 8	7.3 (2.0)	7.6 (2.3)	10.5 (2.4)	2.6 (4.1)	64.63	<0.0001
% change from baseline						
Week 4	5.9%	5.8%	8.6%	1.6%		
Week 8	9.5%	10.0%	13.6%	3.4%		
**MARS**						
Baseline	2.4 (0.7)	2.3 (0.7)	2.4 (0.7)	2.4 (0.7)	0.19	0.900
Change from baseline						
Week 4	−0.3 (0.7)	−0.3 (0.6)	−0.5 (0.5)	−0.2 (0.4)	2.64	0.051
Week 8	−0.4 (0.5)	−0.5 (0.6)	−0.6 (0.6)	−0.2 (0.4)	4.26	0.006
% change from baseline						
Week 4	−12.5%	−13.0%	−20.8%	−8.3%		
Week 8	−16.7%	−21.7%	−25.0%	−8.3%		
**Sleep quality**						
Baseline	6.3 (0.7)	6.3 (0.8)	6.0 (0.9)	6.1 (0.8)	1.96	0.121
Change from baseline						
Week 4	−0.6 (0.5)	−0.8 (0.7)	−1.5 (0.8)	−0.7 (0.5)	23.03	<0.0001
Week 8	−1.3 (0.7)	−1.3 (1.0)	−2.5 (0.8)	−0.8 (0.6)	39.07	<0.0001
% change from baseline						
Week 4	−9.5%	−12.7%	−25.0%	−11.5%		
Week 8	−20.6%	−20.6%	−41.7%	−13.1%		

ARE: Ashwagandha root extract; MLT: melatonin; PLB: Placebo; SD: Standard deviation; SE: Sleep efficiency; MARS: Mental alertness on rising. * One-way analysis of variance (ANOVA). Post hoc Bonferroni’s multiple comparisons (<0.05): **ARE vs. Placebo** (SE: week 4, week 8, change at week 4 and change at week 8; SQ: change at week 8); **ARE/Melatonin vs. ARE** (SE: week 4, week 8, change at week 4 and change at week 8; SQ: week 4, week 8, change at week 4 and change at week 8); **ARE/Melatonin vs. Placebo** (SE: week 4, week 8, change at week 4 and change at week 8; SQ: week 4, week 8, change at week 4 and change at week 8; MARS: change at week 4).

**Table 4:** PSQI and HAM-A scores in the PP dataset.

	ARE (*n* = 48)	MLT (*n* = 47)	ARE–MLT (*n* = 46)	PLB (*n* = 47)	ANOVA	
	Mean (SD)	Mean (SD)	Mean (SD)	Mean (SD)	F	*p* *
**PSQI score**						
Baseline	13.9 (1.7)	14.5 (2.0)	13.6 (1.7)	14.2 (1.7)	2.33	0.076
Change from baseline						
Week 4	−1.8 (0.9)	−2.0 (0.9)	−3.0 (0.3)	−0.8 (0.4)	85.78	<0.0001
Week 8	−2.9 (1.3)	−3.6 (1.6)	−4.7 (0.7)	−1.4 (0.5)	75.21	<0.0001
**HAM-A score**						
Baseline	24.3 (3.3)	24.2 (3.7)	24.7 (3.3)	24.3 (2.6)	0.19	0.901
Change from baseline						
Week 4	−2.5 (1.0)	−2.1 (1.3)	−3.5 (0.8)	−0.9 (0.5)	63.59	<0.0001
Week 8	−4.4 (1.5)	−3.8 (1.9)	−6.0 (1.4)	−1.5 (0.6)	78.49	<0.0001

ARE: Ashwagandha root extract; MLT: melatonin; PLB: Placebo; SD: Standard deviation; PSQI: Pittsburgh Sleep Quality Index; HAM-A: Hamilton Anxiety Rating Scale. * One-way analysis of variance (ANOVA) Post hoc Bonferroni’s multiple comparisons (<0.05): **ARE vs. Placebo** (PSQI: week 4, week 8, change at week 4 and change at week 8; HAM-A: week 8, change at week 4 and change at week 8); **ARE/Melatonin vs. ARE** (PSQI: week 4, week 8, change at week 4 and change at week 8; HAM-A: change at week 4 and change at week 8); **ARE/Melatonin vs. Placebo** (PSQI: week 4, week 8, change at week 4 and change at week 8; HAM-A: week 4, week 8, change at week 4 and change at week 8).

## Data Availability

The raw data supporting the conclusions of this article will be made available by the authors on request.
